# Detection of Organic Compounds in Water by an Optical Absorbance Method

**DOI:** 10.3390/s16010061

**Published:** 2016-01-04

**Authors:** Chihoon Kim, Joo Beom Eom, Soyoun Jung, Taeksoo Ji

**Affiliations:** 1 School of Electronics and Computer Engineering, Chonnam National University, 300 Youngbong-dong, Buk-gu, Gwangju 500-757, Korea; k77chihun@naver.com; 2 Korea Photonics Technology Institute, Medical Photonics Research Center, 9, Cheomdan venture-ro 108beon-gil, Buk-gu, Gwangju 500-779, Korea; jbeom@kopti.re.kr; 3 Samsung Display Co. Ltd., Samsung st. 181, Tangjeong-Myeon, Asan 335-741, Korea; soyounj@yahoo.com

**Keywords:** optical system, absorbance, total organic carbon, multiple linear regression, organic pollutant

## Abstract

This paper proposes an optical method which allows determination of the organic compound concentration in water by measurement of the UV (ultraviolet) absorption at a wavelength of 250 nm~300 nm. The UV absorbance was analyzed by means of a multiple linear regression model for estimation of the total organic carbon contents in water, which showed a close correlation with the UV absorbance, demonstrating a high adjusted coefficient of determination, 0.997. The comparison of the TOC (total organic carbon) concentrations for real samples (tab water, sea, and river) calculated from the UV absorbance spectra, and those measured by a conventional TOC analyzer indicates that the higher the TOC value the better the agreement. This UV absorbance method can be easily configured for real-time monitoring water pollution, and built into a compact system applicable to industry areas.

## 1. Introduction

Water quality monitoring is a key tool in the management of freshwater resources, allowing identification of pollution sources, which can help keep the resources free from contamination by chemicals and microorganisms. As indicators representing the level of subaqueous organics, BOD (biochemical oxygen demand) and COD (chemical oxygen demand) have mainly been employed. However, due to their associated shortcomings as explained below, TOC (total organic carbon) is considered as the most relevant parameter for quantifying the organic pollution in water [1,2,3]. In BOD measurements, errors can be caused by the presence of toxins, non-biodegradable materials, algae, and nitrification. The complicated analysis procedures, and analysis periods of longer than 5 days have also limited the use of the BOD method. Although the COD method is free of the errors resulting from algae and nitrification, and fast to perform (a few hours) compared to BOD testing, it results in hazardous waste, and yields only an approximation of the natural degradation due to the strong oxidant employed [4,5,6,7,8].

In comparison, TOC measurements can be processed more rapidly, without the requirement for extensive amounts of reagents. This is feasible since the TOC analysis is performed by measuring the CO_2_ generated by direct oxidation of the organics. Although the combustion/oxidizing agent method is especially widely used in the U.S. and Germany as an indicator for monitoring the organics in water among the methods of TOC analysis [9], this method encounters problems due to the generation of contamination from the reagents used, and is practically impossible for continuous measurements.

**Figure 1:**
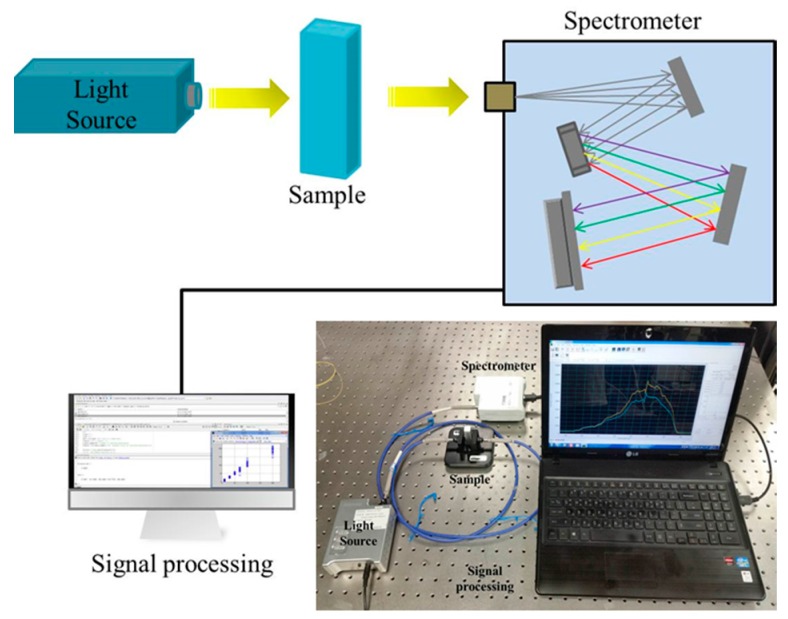
A schematic diagram of the proposed optical system for UV spectrophotometry.

In this paper, in order to overcome the aforementioned problems associated with the combustion TOC method, a UV absorbance-based optical sensor system is proposed for the detection of organic compounds in water. The proposed absorbance method which operates in UV region can monitor TOC in real-time, requiring no chemical pretreatment and thermal reaction. As a result, this method can be easily configured for the detection of water pollutant and applied to industry areas. The schematics and a photograph of the proposed system are shown in Figure 1. The optical system proposed herein adopts an algorithm based upon the analysis of multiple wavelengths, and yields quantitative indicators of the TOC values through the use of a multiple linear regression (MLR) model [10].

## 2. Experimental Section

### 2.1. Working Principles

According to the Beer-Lambert law, when light passes through a material, a certain amount of the light is absorbed. The absorbance can be expressed as the ratio of the incident light and transmitted light intensity, which is related to several material parameters, given by the following equation:

(1) $$\text{A}=\log\frac{{\text{I}}_{0}}{\text{I}}=\text{a}\times \text{b}\times \text{C}$$

where *I_0_* and *I* are the intensity of the incident radiation and the transmitted radiation, respectively, *a* is the absorption coefficient, *b* is the optical path length, and *C* is the concentration. This implies that the concentration of unknown materials dissolved in a solution can be quantified by monitoring the intensity variations of the light due to absorption or scattering, which are caused when the light passes through the material measured [11].

### 2.2. System Configuration and Measurement

The absorbance system employs a deuterium lamp from which continuous light at wavelengths of 200 nm~900 nm is sent into optic fibers with core sizes of 300 μm~1000 μm, and is then passed through test samples placed in a quartz cell (10 × 10 mm^2^). The light coming out of the cell has reduced intensity due to absorbance, and is sent to an optic spectrometer (Scan range: 200 nm~850 nm) through the optic fibers for data analysis.

In order to monitor the organics in water with the absorbance system, potassium hydrogen phthalate (KHP, acidic salt compound) solution at six different concentrations (10, 30, 50, 70, 100, and 200 mg/L) was prepared as a standard sample for TOC testing by dissolving KHP powder (C_8_H_5_KO_4_) in distilled water. The absorbance of the test samples was determined after first measuring dark signals to determine the noise coming from both the spectrometer itself and the reference signals of the DI water. The light intensity that passed through the standard samples was then monitored to yield absorbance values, using the following equation:

(2) $$A=-\log\left(\frac{I_{s}-I_{D}}{I_{R}-I_{D}}\right)$$

where *A* is the absorbance, *I_S_*, *I_R_*, and *I_D_* are the intensity of the sample, the reference, and the dark signal measured, respectively [11,12,13].

## 3. Results and Discussion

**Figure 2:**
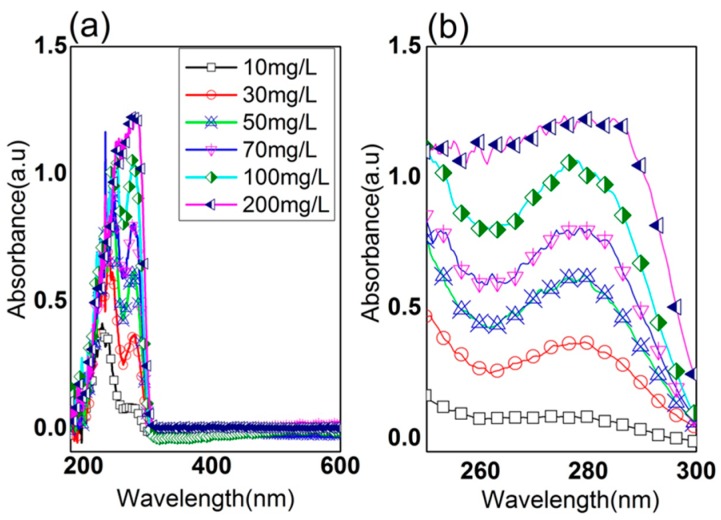
(**a**) UV-Vis spectra of water samples with different potassium hydrogen phthalate (KHP) concentrations; and (**b**) the magnified absorbance spectra in UV range.

The absorbance variations within the range of wavelengths from 200 nm to 900 nm depending on the concentration of the standard solutions are shown in Figure 2a. It can clearly be seen that the variations in the visible range were hardly appreciable, while the absorbance in the UV range varied with increase in the KHP concentration as seen in Figure 2b, thus suggesting the possibility of quantifying the organics. The strong absorbance observed near UV at the wavelength of 200 to 300 nm is known to be due to the specific bonding arrangement in organic molecules, which makes it a good indicator for the presence of conjugated systems, such as those in aromatic molecules [14,15].

An MLR model was adopted to analyze the absorbance signals at several selected wavelengths which appeared to vary in accordance with the concentration of KHP. MLR shows the relationship between a dependent variable (*Y_i_*) and independent variables (*X_ki_*, predictor), as in the formula below:

(3) $$Y_{i}=\beta_{0}+\beta_{1}X_{1i}+\beta_{2}X_{2i}+\cdot \cdot \cdot +\beta_{k}X_{ki}+\varepsilon_{i},\;i=1,\;2,\;\ldots .\;,\;n$$

where ε*_i_* and *β_0_* are the intercept and the error term, respectively. *β_j_*, *j = 0, 1,…, k* are referred to as the regression coefficients of the *j*-th predictors [16].

MLR can model the linear relationship between a dependent variable and one or more independent variables, which involves determining the best set of regression coefficients, *β_j_*, such that the model predicts the experimental values of the dependent variables as accurately as possible. To judge the adequacy of the MLR model to determine whether it fits the observed experimental data, an adjusted coefficient of determination $R^{2}\;(R_{adj}^{2})\;$was explored, which was defined as:

(4) $$R_{adj}^{2}=1-\frac{\frac{SSE}{n-k-1}}{\frac{SST}{n-1}}$$

where *SSE*, *SST*, *n*, and *k* are the error sum of squares, the total sum of squares, the sample size, and the total number of regressors in the linear model, respectively. Since the closer $R_{adj}^{2}$ is to the value 1 the better the model describes the data, the optimal number of independent variables can be determined by taking into account the value of $R_{adj}^{2}$. For MLR analysis, we first selected the UV absorbance at four reliable wavelengths (260, 265, 280, and 285 nm) as the independent variables, whereat the absorbance showed clear variance according to the KHP concentrations, as seen in Figure 2.

The absorbance data collected at the selected wavelengths were analyzed using the stepwise method in the SPSS program. The $R_{adj}^{2}$ values of the four models are given in Table 1. While model 1 involved a single parameter of the wavelength of 265 nm, models 2, 3, and 4 dealt with multiple independent variables; thus, 265 and 280 nm were included in model 2, 265, 280, and 285 nm in model 3, and all four wavelengths were used in model 4 to conduct the MLR analysis.

**Figure 3:**
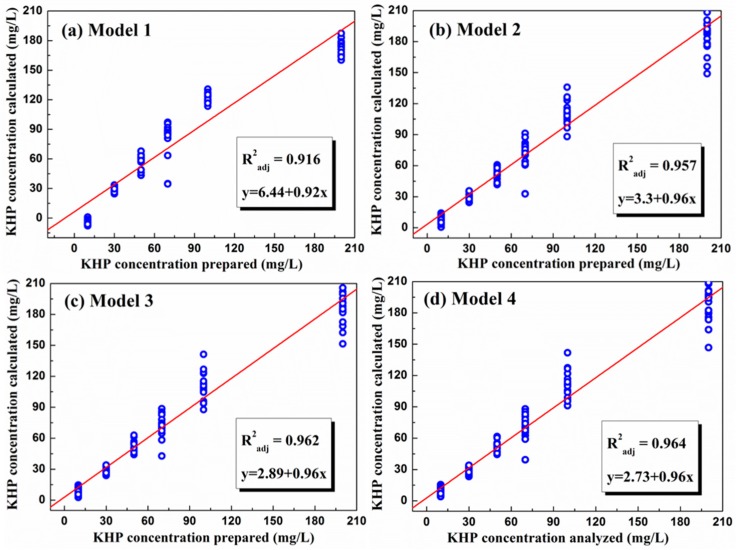
Relationship between the KHP concentration prepared (standard solution) and calculated by multiple linear regression (MLR) analysis for (**a**) model 1; (**b**) model 2; (**c**) model 3; and (**d**) model 4.

As can be seen in the table, the more the independent parameters, the higher the $R_{adj}^{2}$. Thus, the regression analysis with model 4 yielded an $R_{adj}^{2}$ value of 0.964, which implies that the KHP concentrations can be determined with the accuracy of 96.4% from the UV absorption data. Figure 3 shows the relationship between the synthetic KHP concentration and the KHP concentrations calculated by the MLR analysis for the four different models. The optimum regression coefficients extracted by applying model 4 are listed in Table 2, thus indicating that the TOC can be estimated by the following equation:

TOC(mg⁄L) = −1.476 + 207.534(I260) − 236.472(I265) − 199.004(I280) + 112.074(I285)
(5)

where I260, I265, I280, and I286 are the intensities of absorbance at the wavelengths of 260, 265, 280, and 285 nm, respectively.

**Table 1:** Adjusted coefficients of determination of the four different models.

Model	1	2	3	4
$R_{adj}^{2}$	0.916	0.957	0.962	0.964

**Table 2:** Regression Coefficient for Model 4.

$\beta_{0}$	$\beta_{1}$	$\beta_{2}$	$\beta_{3}$	$\beta_{4}$
−1.476	207.534	−236.472	199.004	112.074

**Figure 4:**
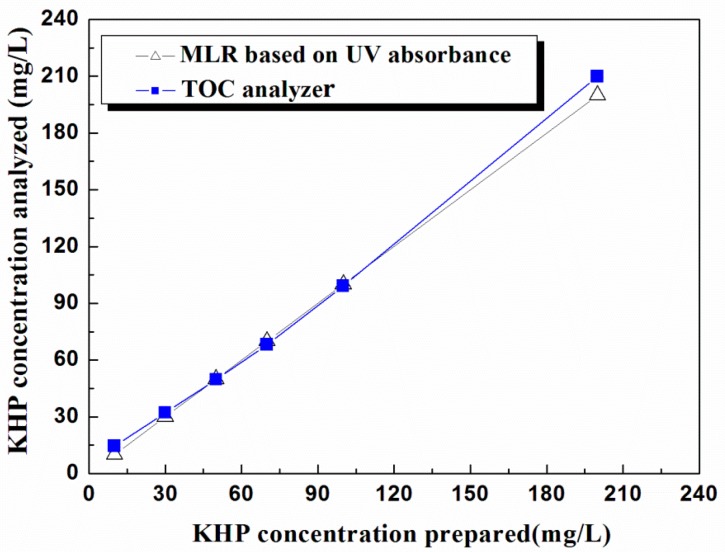
Comparison of KHP concentrations calculated by MLR analysis based on UV absorbance with those measured by a conventional TOC analyzer using the UV-persulfate oxidation method (Multi N/C, Analytik Jena AG, Langewiesen, Germany).

**Figure 5:**
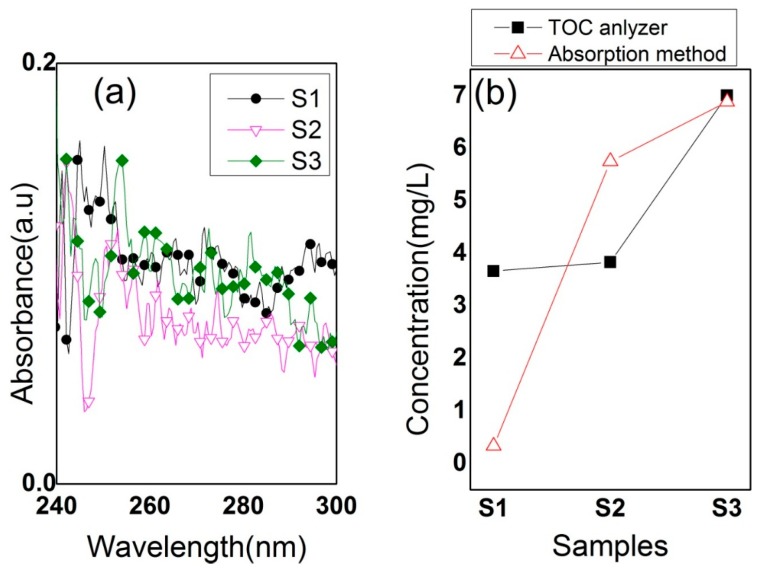
(**a**) UV absorbance spectra for three different real samples (S1: tap water, S2: sea water, and S3: river water). (**b**) Comparison of TOC concentrations based on UV absorbance with those measured by the TOC analyzer instrument. S2 and S3 samples were collected from the South Sea, and the Gwangju stream in Korea, respectively.

In order to evaluate the validity of the MLR model developed herein for correlation of the dissolved concentrations in water and the UV absorbance results, the concentrations calculated by the MLR analysis were compared with those measured by a conventional TOC analyzer (Multi N/C, Analytik Jena AG, Langewiesen, Germany) using the UV-persulfate oxidation method. This comparison, given in Figure 4, clearly shows that the results of the two methods are in good agreement, which strongly supports the possibility of utilizing UV spectrophotometry as a fast and simple means for the monitoring of water quality [17,18]. Moreover, to evaluate the applicability of UV absorbance method to TOC measurements, we prepared three different real samples S1, S2 and S3, which were collected from tap water, sea and river, respectively. Figure 5a,b shows the UV absorbance spectra for the three samples, and the TOC concentration calculated from the UV absorbance spectra in comparison with measured by the TOC analyzer, respectively. The differences in TOC concentrations seen with S1, and S2 are most likely ascribed to the existence of suspended solid in the samples which causes scattering of UV. It should be mentioned that the TOC difference becomes significant as the concentration decreases. Thus, the higher TOC concentration the less influence from the suspended solids. This difference will be minimized by pretreatment of samples using a micro-filter, with which the UV absorbance method could be applicable to a real time water monitoring system for organic compound detection [19,20,21].

## 4. Conclusions

In this paper, we proposed an optical system based on UV spectrophotometry for the detection of organic compounds in water. The optical system proved to be capable of estimating organic carbon content in water in terms of the TOC using the UV absorbance of organic matters. MLR analysis clearly revealed a high degree of correlation between the UV absorbance and TOC concentration, which paves the way for development of a simple and real-time monitoring system for water quality control.
